# Complementary Feeding for Children Aged 6-24 Months: Impact and Maternal Awareness in Al Baha City, Saudi Arabia

**DOI:** 10.7759/cureus.53086

**Published:** 2024-01-28

**Authors:** Khalid Alawad A Mohammed, Elfatih Mirghani M Salih, Fahad Alamr, Mohammed Mousa M Alzahrani, Ahmed Surayyi A Khallufah, Khader Abdullah K Alghamdi, Yahya Fahad Y Alzahrani, Yasir Majhud S Alzahrani

**Affiliations:** 1 Department of Pediatrics, Faculty of Medicine, Al Baha University, Al Baha, SAU; 2 Department of Pediatrics, Dongola University, Dongola, SDN; 3 Department of Pediatrics, Al Baha University, Al Baha, SAU; 4 Faculty of Medicine, Al Baha Univeristy, Al Baha, SAU; 5 Faculty of Medicine, Al Baha University, Al Baha, SAU

**Keywords:** mothers, infant, knowledge, complementary, feeding

## Abstract

Background

Breastfeeding and complementary feeding are essential for baby health and nutrition. Concerning these feeding habits, there is a dearth of information on mother awareness and behaviors in Saudi Arabia. This study intends to evaluate maternal knowledge of breastfeeding and complementary feeding in Al Baha City, Saudi Arabia.

Methodology

This cross-sectional prospective study was conducted among mothers in Al Baha City, Saudi Arabia. Data were collected using a structured electronic questionnaire and analyzed using descriptive statistics and a chi-square test.

Results

We received 524 responses. The majority of participants (358, 68.2%) were found to have sufficient understanding of breastfeeding and complementary feeding. Four hundred and forty participants (84%) were aware that breastfeeding should begin immediately after birth, but only 250 (47.7%) participants knew the proper time to start complementary feeding. Three hundred and ninety-six (75.6%) participants were aware of the proper duration of exclusive breastfeeding, whereas 128 (24.4%) of them did not know. The study showed that factors such as occupation, family economic status, and educational level influence maternal knowledge of complementary feeding.

Conclusions

The study revealed that the majority of participants had good knowledge about breastfeeding and complementary feeding. The study highlights effective training and public awareness initiatives aimed at improving mothers' knowledge and practices regarding feeding. Additionally, it sheds light on the healthcare providers' exemplary knowledge and attitudes toward appropriate feeding practices among mothers in Al Baha City.

## Introduction

A healthy diet is essential for normal children's growth and development. Children's first two years of life represent a critical period for achieving perfect growth and development [1]. The important steps in feeding infants and young children during the first two years of life include the early initiation of breastfeeding, exclusive breastfeeding for the first six months, introducing a suitable and safe complementary diet at the appropriate time, and continuing breastfeeding for up to two years of age or beyond [2]. After the age of six months, breast milk alone is no longer sufficient to meet the nutritional requirements of infants. Consequently, foods and liquids should be introduced, together with breast milk to supply certain nutrients such as protein, iron, zinc, and fat-soluble vitamins [3,4]. The World Health Organization (WHO) recommended exclusive breastfeeding for the first six months of age, followed by the introduction of a nutritionally valuable, age-suitable, and safe complementary diet from six to 24 months that can meet the infant's changing nutritional demands [5].

Complying with dietary requirements during the period of complementary feeding is extremely difficult (infants require a complementary diet with a much higher dietary density than that is required for adults). For example, a breastfed infant at six to eight months requires (per 100 kcal of food) nine times the iron and four times the zinc needed for an adult male [6]. Types of foods that can be started as solid complementary feeding include cereals, fruits, vegetables, and yogurt. Fish and eggs were a bit delayed in being introduced [7]. Regarding allergenic food such as eggs and peanuts, the previous recommendations of practice guidelines were to delay their introduction. However, the new updates recommended early exposure by early introduction of allergenic foods, which effectively diminishes food allergy [8].

Parents should have adequate and proper knowledge regarding how to provide sufficient complementary feeding because parental attitudes, beliefs, and practices about feeding their infants directly affect the nutritional status of their infants [9]. A cross-sectional study carried out to assess the nutritional status of Al Baha elementary male schoolchildren revealed that 10% of Al Baha schoolboys were underweight. In addition, 64% of schoolboys were found to be of normal weight, while 11% were overweight and 15% were obese [10], while another study conducted among intermediate school male Saudi students found that 3.3% were underweight, 70% were normal, 17.3% were overweight, and 9% were obese [11]

We suppose that parents in the Al Baha region lack knowledge regarding proper complementary feeding; therefore, this study aims to assess the level of knowledge of mothers in the Al Baha region about complementary feeding. Low- and middle-income countries bear the greatest impact of incorrect practices in complementary feeding [12]. In light of this, our study also aims to understand the consequences of inadequate supplementary feeding in children aged six to 24 months.

## Materials and methods

Study design

This is a cross-sectional, prospective, community-based study conducted in Al Baha City, Kingdom of Saudi Arabia, from 1/1/2023 to 1/6/2023. An online questionnaire was used to collect data from mothers with children aged 6-24 months. The target population was contacted electronically via social media platforms and other online channels.

Study area

The study was conducted in Al Baha City, the capital city of the Al Baha region, situated in the southwestern region of Saudi Arabia. The city has a population of approximately 500,000 people, and it is known for its mountainous landscape and natural parks.

Sample size and selection criteria

The sample size was calculated using an online website sample size calculator, which determined that 385 participants were needed to achieve a 95% confidence level at a margin of error of ±5%. Using convenient sampling techniques, the study targeted mothers who had children aged six to 24 months or mothers who had experience with complementary feeding.

Inclusion Criteria

Our selection standards focused on mothers with a child aged six to 24 months and those with experience in complementary feeding.

Exclusion Criteria

Mothers who exclusively rely on formula feeding were excluded from the study.

Data collection

An electronic questionnaire was used to collect data, which consisted of two parts. The first part included questions related to the sociodemographic characteristics of the participants, such as age, gender, educational level, occupation, level of income, and number of children. The second part covered the specific objectives of the study, including the awareness of mothers toward complementary feeding and the impact of inadequate complementary feeding. The questionnaire was written in Arabic and distributed to the target population electronically, and the participants had the option to complete it anonymously after signing the informed consent. 

Data analysis

The data were coded and entered into the Statistical Package for IBM SPSS Statistics for Windows, Version 25.0 (IBM Corp., Armonk, NY) for analysis. The collected data were analyzed using descriptive statistics, such as frequencies, percentages, means, and standard deviations. The chi-square test and t-test were used to examine the relationships between the variables and test for significant differences between the variables. A *P*-value of less than 0.05 was considered statistically significant. 

Knowledge score

Participants’ Knowledge Score

The scoring system for knowledge, sourced from a previous study [13], was used to assess maternal knowledge. The aspects used to evaluate knowledge encompassed determining the appropriate time to commence breastfeeding, introducing a supplementary diet, and the recommended duration of exclusive breastfeeding. Additional aspects considered included the types, nutritional components, importance, and consequences of delaying the introduction of a complementary diet. This also encompassed determining the appropriate number of meals for both infants and 1- to 2-year-old toddlers. The correct response scored 1, while the incorrect response scored 0. Mothers’ overall knowledge was categorized into three groups: those with good knowledge (scoring 70% or more), those with fair knowledge (scoring between 50% and 69%), and participants with poor knowledge (scoring less than 50%).

Ethical considerations

The study adhered to ethical principles, and all participants signed the informed consent before being able to proceed to fill out the questionnaire. The form included information about the anticipated benefits of the study, any follow-up procedures, the right to abstain or withdraw from the study at any time, and the right to receive a copy of the informed consent form. The participants were not required to pay for any procedure related to the study. The names of the contributors listed in the proposal were the only authors included in the study.

## Results

We received about 524 complete responses that exceeded the calculated sample size. Table 1 summarizes the sociodemographic characteristics of the participants in the study.

**Table 1:** Sociodemographic factors of the participants (N = 524). Regarding the six illiterate participants, they filled out the questionnaire with the aid of a relative.

Variable		Frequency, *n*	%
Age (Years)	<21	30	5.7
	21-25	67	12.8
	26-30	90	17.2
	31-35	87	16.6
	>35	250	47.7
Nationality	Saudi	509	97.1
	Non-Saudi	15	2.9
Marital status	Married	480	91.6
	Divorced	27	5.2
	Widow	17	3.2
Occupation	Nonemployee	232	44.3
	Government sector employee	216	41.2
	Private sector employee	27	5.2
	Special business	16	3.1
	Student	33	6.3
Educational level	Illiterate	6	1.1
	Primary stage	6	1.1
	Intermediate stage	16	3.1
	High school	76	14.5
	Diploma	59	11.3
	Bachelor	329	62.8
	Postgraduate	32	6.1
Family economic status	Low	16	3.1
	Medium	461	88
	High	47	9
Number of children in the family	1-2	124	23.7
	3-4	182	34.7
	5-6	157	30
	7 or more	61	11.6

**Figure 1:**
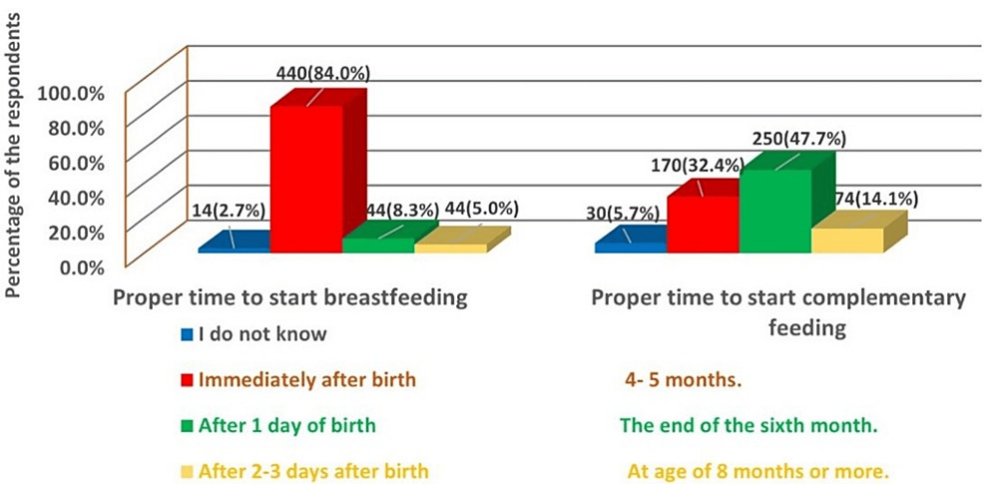
Maternal knowledge regarding proper time to start breastfeeding and complementary feeding (N = 524).

Assessing the knowledge of the participants toward breastfeeding and supplementary feeding revealed that the majority of participants (440, 84%) were aware that breastfeeding should begin immediately after birth but only 250 (47.7%) knew the proper time to start complementary feeding (Figure 1).

Three-quarters of the participants stated that exclusive breastfeeding should continue throughout the first six months of life, whereas 85 (16%) did not know the proper duration of exclusive breastfeeding and 43 (8%) reported it as four to five months. While assessing participants' understanding of the meaning and/or nature of supplementary feeding, 297 (56%) reported that it involves adding semi-solid (gelatinous) and solid food to breastfeeding, while 180 (34%) believed it involves introducing milk formula together with breast milk. Assessing behavior and practices related to breastfeeding and supplementary feeding, approximately 451 (85%) participants said that they used to wash their hands and boil water before preparing meals for their babies. However, 18 (3.4%) and 32 (6.1%) of the mothers, respectively, stated that they did not. The majority of participants (420, 80.2%) were aware that supplementary feeding should consist of an integrated food (proteins, carbohydrates, fatty contents, mineral elements, and vitamins; Table 2).

**Table 2:** Maternal knowledge and practices about breastfeeding and complementary feeding (N = 524). The correct answers are shown in italics.

Item	Responses	Frequency, *n*	%
Duration of exclusive breastfeeding	I do not know	52	9.9
	Till the end of the first month	15	2.9
	2-3 months	18	3.4
	4-5 months	43	8.2
	Till the end of the sixth month	396	75.6
Meaning of supplementary diet	Adding formula milk to breastfeeding	180	34.3
	Adding only semisolid foods to the breastfeeding	47	9
	Adding semisolid and solid foods to breastfeeding	297	56.7
Washing hands before preparing milk or meal	No	18	3.4
	Yes	451	86.1
	Maybe	55	10.5
Boiling water when preparing food or milk	No	32	6.1
	Yes	442	84.4
	Maybe	50	9.5
Complementary feeding should include an integrated food that contains	Proteins	27	5.2
	Sugars	11	2.1
	Fatty contents	5	1.0
	Mineral elements	16	3.1
	Vitamins	45	8.6
	All that was mentioned is true	420	80.2
Overall knowledge	Good	358	68.2
	Fair	140	26.7
	Poor	26	5

**Figure 2:**
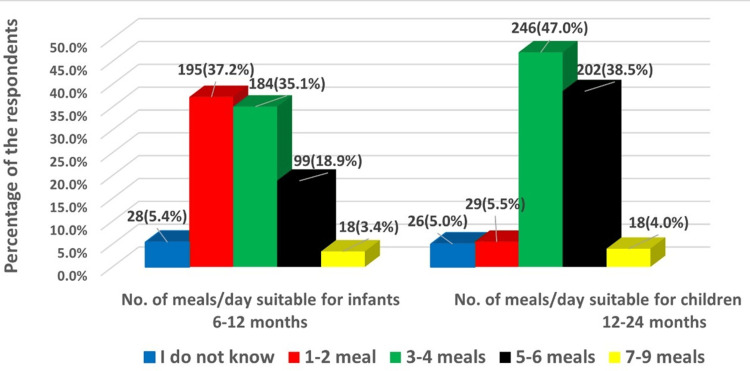
Maternal knowledge concerning the required number of meals for infants aged six to 12 months and toddlers aged 12-24 months.

Regarding the number of meals that infants need at six to 12 months, 195 (37.2%) participants agreed that one to two meals are sufficient. While for children aged 12 to 24 months, 246 (47%) participants thought that three to four meals are adequate (Figure 2).

Overall, the majority of participants have good knowledge about breastfeeding and supplementary feeding (358, 68.2%).

On testing the participants' knowledge about the importance of supplementary feeding for infants, 346 (25.4%) of respondents stated that it is essential for enhancing cognitive development, while 284 (20.8%) believed it is important for preventing diseases, and an equal percentage of 284 (20.8%) referred to its importance in preventing malnutrition. The study revealed that 246 (18%) assumed that complementary feeding enhances physical development, while 204 (15%) believed it contributes to physical growth.

**Figure 3:**
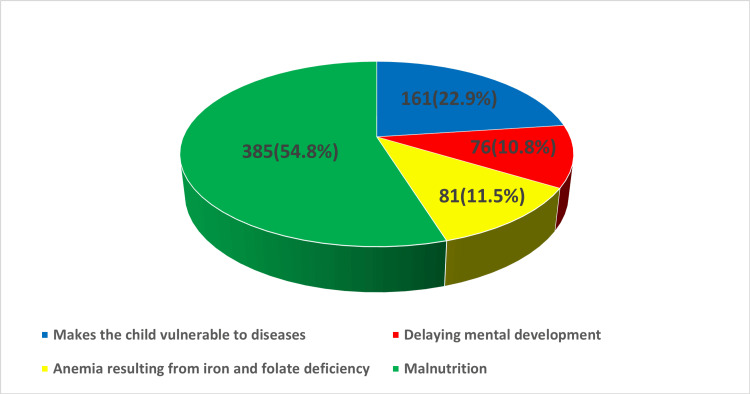
Maternal response regarding consequences of delayed introduction of supplementary feeding. This figure represents a multi-response question.

On assessing the participants' knowledge about the consequences of delaying complementary feeding, majority of the participants (385, 54.8%) believed that delaying complementary feeding could lead to malnutrition, 161 (22.9%) thought it makes the child more vulnerable to disease, 81 (11.5%) stated that it leads to anemia resulting from iron and folate deficiency, while 76 (10.8%) participants reported delaying mental development as a further impact of delayed supplementary feeding (Figure 3). 

**Figure 4:**
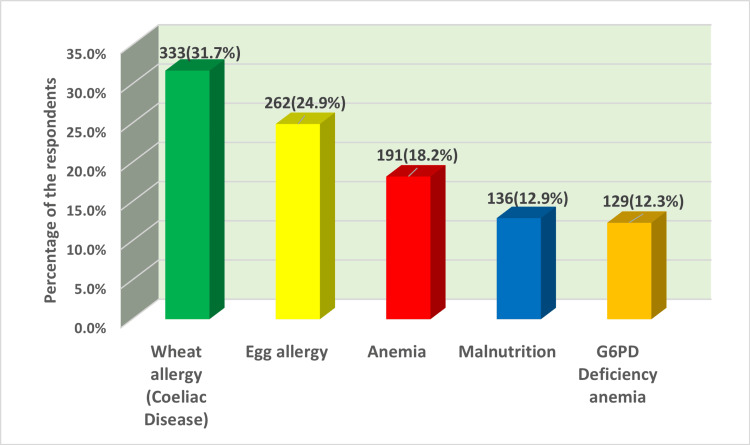
Maternal response regarding diseases that may appear with the introduction of supplementary diet. This figure represents a multi-response question. G6PD, glucose-6-phosphate dehydrogenase

Concerning the negative impact and consequences that may accompany the initiation of complementary feeding, wheat allergy (coeliac disease) and egg allergy were the most commonly known consequences among participants at a percentage of 31.7% (333) and 24.9% (262), respectively. Further reported conditions that may complicate the introduction of complementary feeding include anemia (iron and folate deficiency; 191, 18.2%), malnutrition (136, 12.9%), and glucose-6-phosphate dehydrogenase (G6PD) deficiency anemia (129, 12.3%) (Figure 4).

In attempting to review the causes of delayed introduction of supplementary feeding among mothers, the most prevalent cause was the refusal of the child to accept new types of food (293, 38%). Some mothers stated that they were lacking knowledge (261, 33.9%), while others reported the cause to be the child’s repeated vomiting (159, 20.6%). Some participants also stated that they received incorrect information and notions from various sources regarding the proper time for starting supplementary feeding (33, 4.3%), while 25 (3.2%) claimed that adults (grandma) told them what to do.

On assessing the source of maternal information regarding supplementary feeding, the majority of participants (457, 42.1%) stated that they acquired it from healthcare workers. The second most common source of information was the internet/media (284, 26.2%), followed by family members (274, 25.3%). A lesser proportion of participants obtained knowledge via television (67, 6.1%) and brochures/books for children (3, 0.3%).

Studying the associations between maternal knowledge about breastfeeding and complementary feeding and the sociodemographic factors showed that there were no significant associations between the level of knowledge and age, nationality, marital status, number of family members, child's age, and child's sex. Mothers employed in the private sector exhibited the lowest percentage of adequate knowledge (1, 3.7%), whereas those engaged in special business demonstrated the highest percentage (4, 25%). In terms of family economic status, mothers with a high economic situation had the highest percentage of adequate knowledge (11, 23.4%), while those with a medium economic situation had the lowest percentage (62, 13.4%). There was a noticeable association between educational level and knowledge level evidenced by that the mothers with a higher educational level (postgraduate) had a higher percentage of adequate knowledge (10, 31.3%), whereas illiterate mothers had the lowest percentage (0%) (Table 3).

**Table 3:** Factors associated with mothers' knowledge regarding breastfeeding and complementary feeding.

Factors		Knowledge				*P*-value
		Inadequate		Adequate		
		Frequency, *n*	%	Frequency, *n*	%	
Age	<21	27	90	3	10	0.177
	21-25	63	94	4	6	
	26-30	73	81.1	17	18.9	
	31-35	75	86.2	12	13.8	
	>35	210	84	40	16	
Nationality	Saudi	434	85.3	75	14.7	0.382
	Non-Saudi	14	93.3	1	6.7	
Marital status	Married	409	85.2	71	14.8	0.532
	Divorced	25	92.6	2	7.4	
	Widow	14	82.4	3	17.6	
Occupation	Nonemployee	191	82.3	41	17.7	0.120
	Government sector employee	189	87.5	27	12.5	
	Private sector employee	26	96.3	1	3.7	
	Special business	12	75	4	25	
	Student	30	90.9	3	9.1	
Educational level :	Illiterate	6	100	0	0	0.104
	Primary stage	5	83.3%	1	16.7%	
	Intermediate stage	15	93.8%	1	6.3%	
	High school	67	88.2	9	11.8	
	Diploma	53	89.8	6	10.2	
	Bachelor's	280	85.1	49	14.9	
	Postgraduate	22	68.8	10	31.3	
Family economic status	Low	13	81.3	3	18.8	0.161
	Medium	399	86.6	62	13.4	
	High	36	76.6	11	23.4	
Number of children	1	40	93	3	7	0.552
	2	71	87.7	10	12.3	
	3	66	85.7	11	14.3	
	4	84	80	21	20	
	5	80	85.1	14	14.9	
	6	54	85.7	9	14.3	
	7 or more	53	86.9	8	13.1	

## Discussion

According to our findings, the majority of the participants had good knowledge about breastfeeding and supplementary feeding. Good knowledge is essential for the successful implementation of optimal feeding practices and the avoidance of malnutrition and other related health problems. This contrasts with the findings of previous studies conducted in Unaizah, Saudi Arabia, and Cross River State, Nigeria, which reported a lack of knowledge among mothers regarding complementary feeding [14,15]. However, it aligns with several other studies indicating that the majority of participants demonstrated a high level of knowledge regarding complementary feeding [16-18]. Furthermore, this study's results closely resemble those of a study conducted in Jazan, which revealed that 49% of the participants achieved a high knowledge score [19].

This study revealed several factors that contributed to the delay in supplementary feeding, including the child's refusal of other foods (293, 38%), the mother's misinformation (261, 33.9%), and the child's persistent vomiting (159, 20.6%). These findings are in line with previous research, which revealed comparable reasons for a delayed start to supplemental feeding [20].

In our survey, approximately half of the mothers believed that complementary feeding should commence at six months, with 74 (14.1%) indicating it should start after six months and 30 (5.7%) expressing uncertainty due to a lack of knowledge. This is consistent with the findings of earlier studies [14,21,22] that found 47%, 51.4%, and 51.4% of mothers, respectively, believed that complementary feeding should begin at six months of age. In addition, 396 (75.6%) women in our study knew that exclusive breastfeeding should continue until the end of the sixth month, which is higher than the percentages obtained in an earlier study done in El-Minia, which was 33.6% [23].

Mothers who were employed in the private sector and those whose families were rich had a higher percentage of inadequate knowledge than those mothers who were unemployed or whose families were less wealthy. Mothers with higher education levels were more likely to possess access to sufficient information. The results indicate a significant impact on mothers' education levels, with women holding postgraduate degrees exhibiting the highest knowledge scores, in contrast to 0% for those with no formal education. It is well established that a mother's degree of education is a crucial determinant of her infant feeding practices [24,25].

The majority of the participants in this study acquired knowledge about supplementary feeding from healthcare professionals, followed by the Internet and family members. This was consistent with earlier studies that identified health professionals as the major source of complementary feeding knowledge for mothers [26,27]. In contrast to our finding, Quaidoo reported the online source as the most popular source of information [28], while in a Chilean study, social media was the most common source of information for baby-led weaning [29]. Usually, doctors, nurses, and midwives play a critical role in providing mothers with information and assistance regarding healthy feeding techniques. Therefore, it is essential to ensure that healthcare professionals have received proper training on optimal feeding habits and are equipped with the skills and information necessary to assist mothers in implementing these practices [30].

This study has certain limitations, notably that it was exclusively conducted in Al Baha City, which may restrict the generalizability of the results to the broader population of the entire Al Baha region. The women's responses may be affected by recall bias. Despite these limitations, our research still offers insights into maternal awareness of complementary feeding in Al Baha, providing valuable information for the development of future interventional educational programs.

## Conclusions

This study concluded that the majority of the participants had good knowledge about breastfeeding and complementary feeding. The study reflects the good training and public awareness efforts for enhancing mothers' knowledge and practices toward feeding. Additionally, it emphasizes healthcare providers' comprehensive knowledge and attitudes toward appropriate feeding practices, along with a supportive environment for breastfeeding and complementary feeding among mothers in Al Baha, Saudi Arabia. We identified a deficiency in information regarding the timing of introducing complementary feeding. To address this, there is a need for additional educational campaigns and workshops.
